# Excellent leukemia control after second hematopoietic cell transplants with unrelated cord blood grafts for post-transplant relapse in pediatric patients

**DOI:** 10.3389/fonc.2023.1221782

**Published:** 2023-08-15

**Authors:** Alexandre G. Troullioud Lucas, Jaap Jan Boelens, Susan E. Prockop, Kevin J. Curran, Dorine Bresters, Wouter Kollen, Birgitta Versluys, Marc B. Bierings, Anne Archer, Eric Davis, Elizabeth Klein, Nancy A. Kernan, Caroline A. Lindemans, Andromachi Scaradavou

**Affiliations:** ^1^Department of Pediatrics, Transplantation and Cellular Therapies Service, MSK Kids, Memorial Sloan Kettering Cancer Center, New York, NY, United States; ^2^Department of Stem Cell Transplantation, Princess Máxima Center for Pediatric Oncology, Utrecht, Netherlands; ^3^Department of Pediatrics, Weill Cornell Medicine, New York, NY, United States; ^4^Dana Farber/Boston Children’s Cancer and Blood Disorders Center, Harvard Medical School, Boston, MA, United States; ^5^Division of Pediatrics, University Medical Center Utrecht, Utrecht, Netherlands

**Keywords:** cord blood transplant, relapse, second transplant, leukemia, treatment related mortality

## Abstract

**Background:**

Patients with leukemia relapse after allogeneic hematopoietic cell transplant (HCT) have poor survival due to toxicity and disease progression. A second HCT often offers the only curative treatment.

**Methods:**

We retrospectively reviewed our bi-institutional experience (MSKCC-USA; Utrecht-NL) with unrelated cord blood transplantation (CBT) for treatment of post-transplant relapse. Overall survival (OS) and event-free survival (EFS) were evaluated using the Kaplan-Meier method, treatment-related mortality (TRM) and relapse were evaluated using the competing risk method by Fine-Gray.

**Results:**

Twenty-six patients age < 21 years received a second (n=24) or third (n=2) HCT with CB grafts during the period 2009-2021. Median age at first HCT (HCT1) was 11.5 (range: 0.9-17.7) years and all patients received myeloablative cytoreduction. Median time from HCT1 to relapse was 12.8 (range 5.5-189) months. At CBT, median patient age was 13.5 (range 1.4-19.1) years. Diagnoses were AML: 13; ALL: 4, MDS: 5, JMML: 2; CML: 1; mixed phenotype acute leukemia: 1. Sixteen patients (62%) were in advanced stage, either CR>2 or with active disease. Median time from HCT1 to CBT was 22.2 (range 7-63.2) months. All patients engrafted after CBT. Thirteen patients developed acute GvHD; 7 had grade III or IV. With a median survivor follow-up of 46.6 (range 17.4-155) months, 3-year OS was 69.2% (95% CI 53.6-89.5%) and 3-year EFS was 64.9% (95% CI 48.8-86.4%). Eight patients died, 3 of AML relapse and 5 due to toxicity (respiratory failure [n=4], GvHD [n=1]) at a median time of 7.7 (range 5.9-14.4) months after CBT. Cumulative incidence of TRM at 3 years was 19.2% (95% CI 4.1-34.4%). Notably, all TRM events occurred in patients transplanted up to 2015; no toxicity-related deaths were seen in the 16 patients who received CBT after 2015. Cumulative incidence of relapse was 15.9% (95% CI 1.6-30.2%) at 3 years, remarkably low for these very high-risk patients.

**Conclusions:**

Survival was very encouraging following CB transplants in pediatric patients with recurrent leukemia after first HCT, and TRM has been low over the last decade. CBT needs to be strongly considered as a relatively safe salvage therapy option for post-transplant relapse.

## 1 Introduction

Patients with malignant diseases, who relapse after allogeneic hematopoietic cell transplant (allo-HCT), have poor survival and limited treatment options. A second transplant often represents the only potentially curative approach (1, 2). Historically outcomes of second transplants have been discouraging due to disease progression and toxicity. However, with current treatment advances and better graft choices results of second transplants have improved over the recent years (3, 4).

Since patients who relapse after HCT have very high-risk disease a graft with potent antileukemic activity is preferred. Unrelated cord blood (CB) grafts have shown strong graft-versus-leukemia effect after first allo-HCT (HCT1), particularly in patients with AML and minimal residual disease (MRD) (5–7) or even refractory disease (8). Based on the clinical experience as well as preclinical data there is growing evidence of the unique immunological properties of CB T cells (9, 10) making these grafts ‘intrinsically’ more effective as graft-versus-leukemia treatment (11). As a result, CB grafts, offering both strong antileukemic properties and prompt availability, would be the graft of “choice” for patients with high-risk malignant disease (12), including those undergoing second allo-HCT. Additional advantages include no risk to a related or unrelated donor (13) and the possibility of selecting specific HLA alleles for tumor antigen recognition in cases that relapse is a result of immune escape (14).

We hypothesized that second transplants with CB grafts for patients with hematologic malignancies who relapsed after first HCT represent a feasible option, and their outcomes have improved with current treatment advances, as have those of other graft sources (3, 4). We describe our bi-institutional experience using unrelated CB grafts for treatment of post-transplant relapse in 26 pediatric patients.

## 2 Materials and methods

### 2.1 Data collection

An Institutional Review Board (IRB)-approved retrospective analysis of data was performed on patients younger than 21 years, who received a subsequent allo-HCT with a CB graft for relapse at Memorial Sloan Kettering Cancer Center (New York, USA) and the University Medical Center Utrecht/Princess Máxima Center for Pediatric Oncology (Utrecht, the Netherlands) during the period 2009 to 2021. All follow-up data are as of November 1, 2022. Survivors had at least 1 year of follow-up. Patients were included in this analysis irrespective of conditioning intensity, previous transplant donor source, timing after previous HCT, underlying disease, co-morbidities, etc. Supportive care and GvHD prophylaxis were per institutional guidelines.

### 2.2 Outcomes

Main outcomes of interest were overall survival (OS), treatment related mortality (TRM) and relapse. OS time was defined as time from the CB transplant (CBT) to time of death from any cause or to time of last follow-up for survivors. TRM was defined as death by any cause other than relapse. Relapse was diagnosed by bone marrow or peripheral blood evaluation.

Other outcomes of interest included time to neutrophil and platelet recovery, development of acute graft versus host disease (aGvHD) and event-free survival (EFS). Engraftment day was defined as the first of three consecutive days with an absolute neutrophil count (ANC) greater than 0.5 x 10^9/L. Graft failure after CBT was defined by either no engraftment at day 42 or loss of the graft after initial engraftment (secondary graft failure). Platelet recovery was defined as the first day of platelet count greater than 20 x 10^9/L without transfusion support for 7 consecutive days. Acute GvHD was defined by CIBMTR criteria. EFS was evaluated with events defined as graft failure, relapse or death for any reason. Surviving patients were censored at the date of last contact.

### 2.3 Statistical analysis

The Kaplan-Meier method was used to analyze OS and EFS. For analysis of cumulative incidences of TRM, relapse and aGvHD the Fine-Gray competing risk method was used. All statistical analyses were done using R statistical software, version 4.2.1, packages: tidyverse, survival, survminer, prodlim, cmprsk.

## 3 Results

Twenty-six patients received a second (n=24) or a third (n=2) HCT with a CB graft (**Table 1**).

**Table 1:** Patient and transplant characteristics.

Patient	Gender	HCT1					Cord blood transplant													
		Conditioning	Donor (R/U)	Graft	Time to relapse (mo)	Time HCT1-CBT (mo)	Age (years)	Tx Year	Diagnosis	Disease Status	Conditioning	GvHD PPx	CB Graft	HLA Match	ANC > 500 (days)	Plts > 20k (days)	aGvHD	Patient Status	Relapse	Follow up (mo)
1	M	TBI1200/VP16	U	BM	55.7	59.6	13.3	2012	ALL	CR2	Bu/Flu/Clo	CsA/Pred/MMF	Double	6/6, 6/6	20	38	Grade 2	Deceased	No	7.7
2	M	Bu/Flu	U	BM	5.5	7.0	1.4	2009	JMML	active disease	Treo/Flu/Mel	CsA/Pred	Single	6/6	18	32	Grade 3	Alive	No	155.0
3	F	Bu/Flu	U	CB	22.5	25.6	19.1	2013	AML	CR3	Flu/Cy/TBI400	CsA/Pred/MMF	Single	4/6	19	69	No	Deceased	No	6.0
4	F	Bu/Flu/Clo	U	CB	20.2	22.1	14.6	2015	AML	CR3	Flu/Cy/TBI400	CsA/MMF	Single	4/6	26	44	No	Deceased	Yes	3.1
5	M	Bu/Flu/Clo	U	CB	10.5	13.2	14.5	2017	AML	CR3	Treo/Flu/TBI400	CsA/Pred	Single	4/6	13	27	Grade 1	Alive	No	64.1
6	F	Bu/Flu/Clo	U	BM	10.7	13.5	7.2	2018	MDS	stable disease	Treo/Flu/TT	CsA/Pred	Single	5/6	18	57	Grade 3	Alive	No	47.9
7	F	Bu/Flu/Clo	R	BM	29.0	31.6	10.0	2021	MDS	stable disease	Treo/Flu/TT	Tacro/Pred	Single	4/6	14	39	No	Alive	No	21.1
8	M	Treo/Flu/TT	R	BM	10.0	16.1	11.8	2018	MDS	stable disease	Bu/Flu/Clo	CsA/Pred	Double	4/6, 4/6	34	50	No	Alive	Yes	51.9
9	M	Bu/Flu/Clo	U	BM	40.1	42.2	15.9	2018	secMDS/AML	CR1	Treo/Flu/TBI400	CsA/Pred	Single	5/6	21	45	Grade 2	Alive	No	50.7
10	F	Bu/Flu/Clo	U	BM	20.0	22.6	14.6	2019	AML	CR2	Treo/Flu/TT	CsA/MMF	Single	5/6	18	40	No	Alive	No	39.0
11	M	Bu/Flu/Clo	R	BM	9.0	25.9	14.8	2019	AML	CR4	Treo/Flu/TT	CsA/Pred	Single	5/6	16	33	No	Alive	No	40.1
12	M	Bu/Flu/TT	U	CB	10.8	18.6	17.7	2020	AML	CR4	Treo/Flu/TT	CsA/Pred	Single	4/6	17	46	Grade 2	Deceased	Yes	9.5
13	M	Bu/Flu/Clo	U	CB	5.6	8.5	13.7	2020	AML	CR3	Treo/Flu/TT	Tacro/Pred	Single	4/6	24	33	No	Alive	No	30.2
14	M	Bu/Flu/Clo	U	CB	30.0	42.0	12.3	2020	ALL	CR4	TBI1200/VP16	CsA/Pred	Single	4/6	26	24	No	Alive	No	30.0
15	M	Mel/TT/Flu	R	PB	7.6	18.8	13.3	2015	AML	active disease	Cy/Flu/TT/TBI400	CsA/MMF	Double	4/6, 4/6	20	50	Grade 2	Deceased	No	14.3
16	M	TBI1200/Cy	R	PB	14.8	23.8	7.8	2018	secMDS/AML	active disease	Clo/Mel/TT	Tacro/MMF	Single	5/6	19	27	Grade 4	Alive	No	46.9
17	M	Bu/Mel/Flu	U	PB	9.7	11.9	15.8	2015	AML	CR2	Cy/Flu/TT/TBI400	CsA/MMF	dCB/Haplo	4/6, 4/8	11	51	Grade 3	Deceased	No	5.9
18	F	Bu/Mel	R	BM	29.7	33.1	17.5	2017	AML	CR2	Clo/Mel/TT	CsA/MMF	Double	4/6, 4/6	17	36	No	Alive	No	48.2
19	F	Clo/Mel/TT	U	CB	5.5	22.3	2.9	2011	AML	CR3	Flu/Cy/TBI1200	CsA/MMF	Double	5/6, 4/6	21	33	No	Deceased	Yes	7.9
20	F	TBI/Cy	R	BM	10.8	15.7	9.0	2012	ALL	CR3	Clo/Mel/TT	Tacro/MMF	Double	5/6, 4/6	21	50	No	Alive	No	104.2
21	M	Clo/Mel/TT	U	BM	10.8	22.1	3.0	2012	JMML	active disease	Flu/Dauno/TBI1320	CsA/MMF	Double	5/6, 5/6	40	153	Grade 3	Deceased	No	10.1
22	M	Bu/Flu/Mel	R	PB	5.9	16.8	18.7	2019	MPAL	CR2	Cy/Flu/TBI1375	CsA/MMF	Single	4/6	15	26	Grade 3	Alive	No	39.4
23	M	Cy/TT/TBI1500	U	PB	16.1	50.9	9.8	2021	ALL	CR4	Clo/Mel/TT	CsA/MMF	Single	5/6	17	38	Grade 3	Alive	No	19.9
24	F	Bu/Flu/Clo	R	BM	6.2	9.9	18.6	2021	AML	CR2	Cy/Flu/TT/TBI400	Tacro/MMF	Single	4/6	21	43	No	Alive	No	17.4
25	M	VP16/Cy/TBI	R	BM	21.0	26.9	12.4	2014	CML	CR4	Treo/Flu/TBI400	CsA/Pred	Single	5/6	23	99	No	Alive	No	96.1
26	F	Treo/Flu/TT	U	BM	36.0	63.2	16.5	2019	AML	CR3	TBI1200/Cy	CsA/Pred	Single	6/6	17	101	No	Alive	Yes	45.8

male (M), female (F), hematopoietic cell transplant (HCT), total body irradiation (TBI), etoposide (VP16), busulfan (Bu), fludarabine (Flu), clofarabine (Clo), treosulfan (Treo), thiotepa (TT), melphalan (Mel), cylcophosphamide (Cy), unrelated (U), related (R), bone marrow (BM), cord blood (CB), peripheral blood (PB), cord blood transplant (CBT), acute lymphoblastic leukemia (ALL), juvenile myelomonocytic leukemia (JMML), acute myleoid leukemia (AML), myelodysplastic syndrome (MDS), secondary MDS/AML (secMDS/AML), mixed phenotype acute leukemia (MPAL), chronic myeloid leukemia (CML), complete remission (CR), daunorubicin (Dauno), graft versus host disease prophylaxis (GvHD PPx), cyclosporine (CsA), prednisolone (Pred), mycophenolate mofetil (MMF), tacrolimus (Tacro), haploidentical transplant (Haplo), double cord blood (dCB), absolute neutrophil count (ANC), platelets (Plts), acute graft versus host disease (aGvHD).

### 3.1 Patient and first allo-HCT characteristics

Median age at HCT1 was 11.5 (range 0.9-17.7) years. Diagnoses were acute myeloid leukemia (AML; n=13 [50%]), acute lymphoblastic leukemia (ALL; n=6 [23.1%]), myelodysplastic syndrome (MDS; n=3 [11.5%]), juvenile myelomonocytic leukemia (JMML, n=2 [7.7%]), chronic myelogenous leukemia (CML; n=1 [3.8%]) and mixed phenotype acute leukemia (MPAL; n=1 [3.8%]). All patients received myeloablative cytoreduction. Donors were related (n=10 [38.5%]) or unrelated (n=16 [61.5%]). Graft sources included bone marrow (BM; n=14 [53.8%]), *ex vivo* T cell depleted peripheral blood (PB; n=5 [19.2%], or CB (n=7 [26.9%]).

Median time from HCT1 to relapse was 12.8 (range 5.5-189) months. There was no significant difference in time to relapse after HCT1 between patients who had related versus unrelated donors (median time to relapse 12.8 and 13.4 months, respectively).

### 3.2 Cord blood transplant characteristics

At the time of the CBT, median patient age was 13.5 (range 1.4-19.1) years. Two patients who had ALL at HCT1 subsequently developed secondary MDS, which was the reason for the second transplant. Overall, 16 patients (61.5%) were in advanced stage, either CR>2 or with active disease (n=4) at time of CBT. CB units were ≥4/6 HLA-matched to patients. Median time from HCT1 to CBT was 22.2 (range 7-63.2) months.

Various conditioning regimens were used, as shown in the **Table 1**. Briefly, 13 patients received chemotherapy-only cytoreduction, while the other 13 had total body irradiation (TBI) also; of those, 8 received reduced dose TBI (400 cGy) and the remaining had full dose (TBI dose ≥1200 cGy).

For two patients (patient 25 and 26) we describe the outcomes of their third allo-HCT, with a CB graft. Briefly, patient 25 received a CB graft for relapse after HCT1 but failed to engraft – a third HCT with another CB was administered. Patient 26 had a BM transplant for relapse after HCT1 but relapsed shortly afterwards and then received a CB graft. Both received myeloablative cytoreduction with busulfan, fludarabine and clofarabine for their second transplant.

### 3.3 Outcomes

All patients achieved engraftment. Median time to ANC>500 was 19 (range: 11-40) days, median time to platelet recovery was 41.5 (range 24-153) days. Twelve patients developed aGvHD after CBT, 7 of those had grade III or IV (cumulative incidence at 100 days 46.2% and 26.9%, respectively).

**Figure 1:**
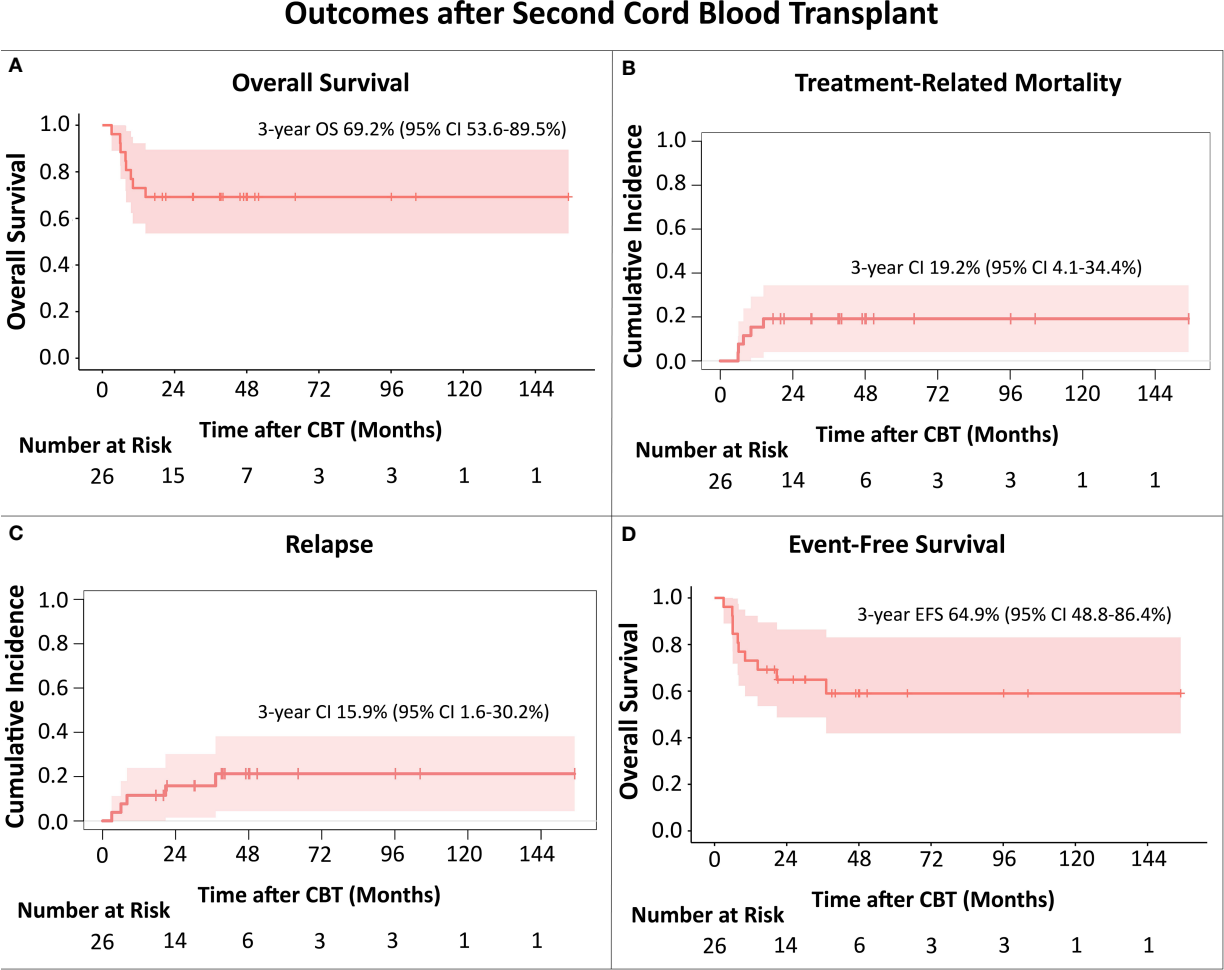
**(A)** Overall Survival (OS); **(B)** Treatment-Related Mortality (TRM); **(C)** Relapse; **(D)** Event-Free survival (EFS). The Kaplan-Meier method was used to analyze OS and EFS. For analysis of cumulative incidences of TRM and relapse the Fine-Gray competing risk method was used. All TRM events occurred in patients transplanted up to 2015.

Of the 26 patients, 18 remain alive with a median follow-up time of 46.6 (range 17.5-155) months, including two of four patients that underwent CBT in active disease. OS at 3 years was 69.2% (95% CI 53.6-89.5%; **Figure 1A**).

Eight patients died after the CB transplant. For three, the cause of death was relapse, all had AML, 2 were in CR3, one in CR4 prior to CBT, and relapses were at 3, 6 and 8 months, respectively. Five patients died of toxicity, respiratory failure (n=4), GvHD with multi-organ failure (n=1), at a median time of 7.7 (range 5.9-14.4) months after CBT. The cumulative incidence of TRM at 3 years was 19.2% (95% CI 4.1-34.4%; **Figure 1B**). Notably, all TRM events occurred in patients transplanted up to 2015 (5 out of the 10 CB recipients), with no toxicity-related deaths in the 16 patients who received CB transplant after 2015.

There were 5 patients with hematologic relapse after CBT (**Table 1**). Three patients with early relapses died of disease progression, while two patients, who relapsed at 20.7 months (patient with AML, third allo-HCT) and 37 months (patient with MDS) remain alive for 25 and 15 months, respectively, with ongoing targeted maintenance therapy. Cumulative incidence of relapse at 3 years was 15.9% (95% CI 1.6-30.2%; **Figure 1C**). 3-year EFS was 64.9% (95% CI 48.8-86.4%; **Figure 1D**). In this relatively small cohort, there was no difference in survival for patients that relapsed earlier than 12 months after HCT1 versus those with later relapses (p=0.462).

## 4 Discussion

Our contemporary bi-institutional analysis shows very encouraging survival after second (third in two patients) allo-HCT with CB grafts for post-transplant relapse, with a 3-year OS of nearly 70%. While we describe a 3-year cumulative incidence of TRM of 19.2%, the deaths were in patients transplanted during the period 2009-2015. It is reassuring that there have been no toxicity-related deaths in the 16 recipients who received CB transplants during the most recent period (2016–2021). Furthermore, the 3-year cumulative incidence of relapse of 15.9% was remarkably low considering this very high-risk group. This underscores the strong graft-versus-leukemia potential of the CB grafts even in patients with post-transplant relapse.

These data show superior outcomes to those reported in the CIBMTR analysis of 251 children, adolescents and young adults with acute leukemia, who received second allo-HCT for relapse (15). In that analysis, 2-year leukemia-free survival was only 33% and survival after CBT (n=83) was lower than after HCT with HLA-identical siblings or matched unrelated donors, as TRM was significantly higher with CBT. However, the CIBMTR study evaluated transplants performed during the period 2001-2014, with 74% of the second HCT taking place before 2010, and, therefore, do not reflect current approaches. Improvements in preparative regimens to decrease toxicity and supportive care measures have addressed several of the reasons for prior treatment failures. As a result, two large studies including 221 pediatric patients with acute leukemia/MDS who received second HCT at St. Jude’s Children’s Research Center (3) and 122 children with AML treated in Europe (I-BFM study) (4), showed significant improvement in the probability of survival when the second HCT with BM or PB graft was performed after 2010.

For CBT specifically, omitting ATG from the conditioning regimens has resulted in faster immune reconstitution, lower rates of viral reactivation, and reduction in TRM (16, 17). While our relatively small number of patients does not allow evaluation of specific cytoreduction regimens, chemotherapy-only regimens (18), low dose TBI, and lower toxicity agents such as treosulfan (19, 20) have very likely contributed to the improved outcomes. In addition to changes in cytoreduction, CB graft selection has been focusing on higher TNC/CD34 cell doses and allele level HLA matching (21, 22). With these optimizations, mortality after first allo-HCT with CB has decreased significantly and overall outcomes have improved (6, 12, 23, 24). In fact, a recent analysis of 317 pediatric patients with AML treated at US and European transplant centers showed no difference in non-relapse mortality among the three graft sources: HLA-identical sibling, matched unrelated donor or (single) CB graft (25). Although limited data exist for second transplants with CB grafts, our results are in agreement with a recent UK study evaluating outcomes in children with relapsed/refractory AML transplanted during recent years (2014–2021); in that analysis the 2-year EFS of the 24 patients who received a second transplant with a CB graft was 69% (95% CI 45-84%) (8).

Although our outcomes are important to report, we acknowledge our study’s limitations: it is a retrospective analysis, with small number of patients and various preparative regimens, without comparison to other graft sources. However, the results indicate fewer toxicity-related deaths in the recent period, and low incidence of relapse, highlighting the importance of including CBT in clinical trials for second transplants after relapse.

Additionally, in our limited cohort, patients with more advanced disease (>CR2) did not have worse survival than patients in earlier remission. Further, in contrast to older studies, time of relapse after HCT1 did not seem to affect outcomes after HCT2. Finally, two of the four patients who were transplanted with active disease remain alive and in remission indicating the strong anti-leukemic potential of the CB graft even in refractory myeloid leukemia. In support of this finding, the UK CBT analysis showed 2-year EFS of 44.8% in 23 children with relapsed refractory AML and a recent Japanese study reported higher survival after CB compared to haplo-grafts in adults with refractory AML (8, 26).

In summary, our bi-institutional data show very encouraging outcomes after second allo-HCT with CB grafts in pediatric patients who relapsed after first-HCT, especially for transplants performed during the recent period. Considering the potent antileukemic activity, particularly evident in high-risk disease, and the prompt graft availability, we strongly recommend that CBT be considered as one of the options in the setting of post-transplant relapse, preferably in trials using standardized regimens to study the benefit of CBT prospectively.

## Data availability statement

The raw data supporting the conclusions of this article will be made available by the authors, without undue reservation.

## Ethics statement

Ethical review and approval was not required for the study on human participants in accordance with the local legislation and institutional requirements. Written informed consent to participate in this study was provided by the participants’ legal guardian/next of kin.

## Author contributions

All authors provided contributions to the concept or design of the work; or the acquisition, analysis, or interpretation of data for the work. ATL, AS and CL wrote the initial drafts and all authors provided critical review for intellectual content. All authors approved the submitted version.
